# Global Fast Terminal Sliding Mode Control for Trajectory Tracking Control of Quadrotor UAVs

**DOI:** 10.3390/s25247480

**Published:** 2025-12-09

**Authors:** Runze Gao, Shaobo Wu, Hongguang Li

**Affiliations:** Xi’an Institute of Applied Optics, Xi’an 710065, China; grz990113@163.com (R.G.); redlight007@163.com (H.L.)

**Keywords:** quadrotor UAV, terminal sliding mode, trajectory tracking, rapidity, disturbance rejection

## Abstract

**Highlights:**

**What are the main findings?**

- To address the trajectory tracking control problem of quadrotor UAVs, a trajectory tracking control method based on the GFTSMC algorithm is proposed.

**What are the implications of the main finding?**

- The proposed GFTSMC algorithm can enhance the rapidity of trajectory tracking for quadrotor UAVs and strengthen the anti-disturbance capability of the quadrotor UAV system.

**Abstract:**

A fast and stable flight control system is crucial for improving the efficiency of unmanned aerial vehicle (UAV) missions. Focusing on the trajectory tracking control of quadrotor UAVs, this paper proposes a trajectory tracking control method based on the global fast terminal sliding mode control (GFTSMC) algorithm to address the slow response speed and insufficient anti-disturbance capability inherent in the widely used Proportional–Integral–Derivative (PID) control algorithm and conventional sliding mode control (SMC) algorithm. Firstly, considering the gyroscopic moment of a quadrotor UAV’s rotors, an accurate kinematic and dynamic model of a quadrotor UAV is established, and the trajectory tracking problem faced by such UAVs is decoupled into the command tracking problems of the position loop and the attitude loop. Secondly, GFTSMC controllers are designed for these loops, and the Lyapunov principle is adopted to prove the stability of the designed controllers. Finally, simulation verification is carried out. The simulation results show that, compared to PID control, GFTSMC-based trajectory tracking control for quadrotor UAVs exhibits the characteristics of no overshoot, higher tracking accuracy, and stronger anti-disturbance capability. Compared to nonsingular terminal sliding mode control (NTSMC) and SMC, GFTSMC-based trajectory tracking control reduces the steady-state convergence time by 33.8% and 36.5% and the steady-state disturbance error by 83.1% and 97.3%, respectively, demonstrating faster response speed and stronger anti-disturbance capability. Therefore, the application of GFTSMC significantly improves the trajectory tracking control performance of quadrotor UAVs, thereby supporting them in performing operations in scenarios requiring high real-time performance, precision, and anti-disturbance capability.

## 1. Introduction

With the continuous iteration and evolution of robotics technology, its application value and importance in human production and daily life have become increasingly prominent [1]. As a key branch of aerial mobile robots, UAVs can be equipped with various types of sensors to provide efficient and accurate technical support and solutions for many fields such as equipment status inspection [2], cultural heritage protection [3], and search and rescue [4]. Quadrotor UAVs have been widely used as UAV platforms due to their advantages including agile maneuverability, vertical take-off and landing, and simple operation [5]. When performing operational tasks, quadrotor UAVs often need to track a predefined route; thus, achieving fast, accurate, and stable trajectory tracking control is a core objective in the design of quadrotor UAV flight control algorithms. However, the quadrotor UAV system exhibits strong nonlinearity, multivariable coupling, and high susceptibility to external disturbances, which pose numerous technical challenges to research on trajectory tracking control. Driven by these complex control requirements, trajectory tracking control technology for quadrotor UAVs has received significant attention from the academic community in recent years [6].

To address the trajectory tracking control problem faced by quadrotor UAVs, researchers have proposed a variety of control methods. Currently, the PID control method is the most widely used in quadrotor UAV engineering applications. Traditional PID control offers advantages such as a simple structure and ease of implementation; however, when the control system is subjected to external disturbances or parameter perturbations, PID control suffers from low tracking accuracy and poor stability [7]. The authors of Reference [8] proposed an adaptive PID control algorithm that enhances the robustness of the control system. Tiep [9] applied fuzzy control to adjust the parameters of the quadrotor UAV PID controller in real time for trajectory tracking. This approach not only ensured the optimal dynamic performance of the UAV but also effectively reduced its trajectory tracking error. A nonlinear model of the vehicle was adopted for mass identification; specifically, the weighted recursive least squares (WRLS) method was employed for online identification to tune the PID controller, which effectively reduced the adverse effects of noise and external disturbances on the system [10]. Although the control schemes in these studies improve the stability of UAV trajectory tracking to a certain extent, with the increase in the complexity of trajectory tracking, the impact of the UAV’s nonlinear characteristics on system stability becomes more significant—this leads to notable degradation in the trajectory tracking performance of linear control methods such as PID control [11].

As a nonlinear control algorithm, SMC exhibits excellent robustness and has been widely applied in flight control systems in recent years [12]. The authors of Reference [13] introduced an adaptive switching gain into the control law of SMC and proposed an adaptive sliding mode control method, which achieves fast adaptation to parameter uncertainties and external disturbances as well as ensures robustness in UAV navigation and control. The control scheme designed in Reference [14] combined SMC with backstepping control, enhancing the rapidity and robustness of the latter. Notably, SMC not only improves the robustness of UAV systems but also endows them with favorable capability against external disturbances. In Reference [15], an SMC controller that enhanced the robustness of UAV systems subjected to bounded disturbances was developed. A dual-loop control system was proposed in Reference [16], where a backstepping sliding mode controller was designed for the inner loop, while an integral sliding mode controller was constructed for the outer loop—this configuration ensured the position tracking capability of quadrotors even under severe, uncertain disturbances. The authors of Reference [17] designed an SMC controller for UAV systems subjected to external disturbances and parameter uncertainties, achieving excellent tracking performance in both simulations and flight tests. In terminal sliding mode control (TSMC) [18,19], the linear sliding mode surface is formulated as a nonlinear function. This modification not only ensures the finite-time reaching of the sliding mode surface but also guarantees the finite-time convergence of system outputs, thereby improving the convergence speed of SMC. The global fast terminal sliding mode controller proposed in Reference [20] enabled UAV systems to track the desired position within finite time. The authors of Reference [21] proposes an adaptive fast nonsingular terminal sliding mode control scheme. By integrating fast nonsingular terminal sliding mode control with adaptive estimation technology, it effectively improves the system’s tracking performance and enhances the chattering suppression effect. The authors of Reference [22] applied the TSMC algorithm to both the position loop and attitude loop, and integrated a sliding mode disturbance observer with the TSMC controller of the attitude loop, which strengthened the system’s capability against external disturbances. A sliding mode observer was also introduced in Reference [23], ensuring the system’s anti-disturbance capability and finite-time convergence. In Reference [24], neural networks were combined with TSMC and used to approximate unknown quantities, which not only improved the system’s robustness against uncertainties but also enhanced the system’s speed in tracking the desired attitude. To address the attitude control challenge of quadrotor UAVs under uncertainties and external disturbances, Kahouadji, M. [25] proposed a Super Twisting control scheme, which aims to reduce control effort and improve trajectory tracking accuracy.

This paper focuses on the trajectory tracking control problem faced by quadrotor UAVs and proposes a trajectory tracking control scheme based on the GFTSMC algorithm. Firstly, a dual-closed-loop control model consisting of the position loop and attitude loop of quadrotor UAVs is established, which transforms the trajectory tracking problem into a command tracking problem for these loops. During the modeling process, the gyroscopic moment of the UAV rotors is taken into account, resulting in a more accurate model. Secondly, the terminal sliding mode control (TSMC) algorithm is improved by incorporating double power terms into both the terminal sliding mode surface and the reaching law. On this basis, global fast terminal sliding mode controllers are constructed for the position loop and attitude loop. Additionally, the Lyapunov method is employed to prove the stability of the proposed controllers. Finally, comparative numerical simulation experiments are conducted, the results of which verify the effectiveness and superiority of the proposed control algorithm. The main contributions of this paper are as follows: the GFTSMC controller improves the rapidity of trajectory tracking for quadrotor UAVs, enhances the anti-disturbance capability of the quadrotor UAV system, and ensures faster and more stable trajectory tracking control. The saturated function is adopted to replace the sign function in the switching term, which ensures the continuity of the control input and reduces the chattering generated during the control process.

## 2. Quadrotor Dynamics Modeling and Control Framework

To achieve high-fidelity trajectory tracking, we first establish a six-degree-of-freedom (6-DoF) dynamics model of the quadrotor, explicitly accounting for gyroscopic effects in the rotors. Then, the trajectory tracking control problem facing the quadrotor UAV is transformed into the tracking control problem of the position loop and the attitude loop in a double-closed-loop control system, establishing an overall control scheme for the quadrotor UAV.

### 2.1. Modeling of Quadrotor Unmanned Aerial Vehicle

**Figure 1:**
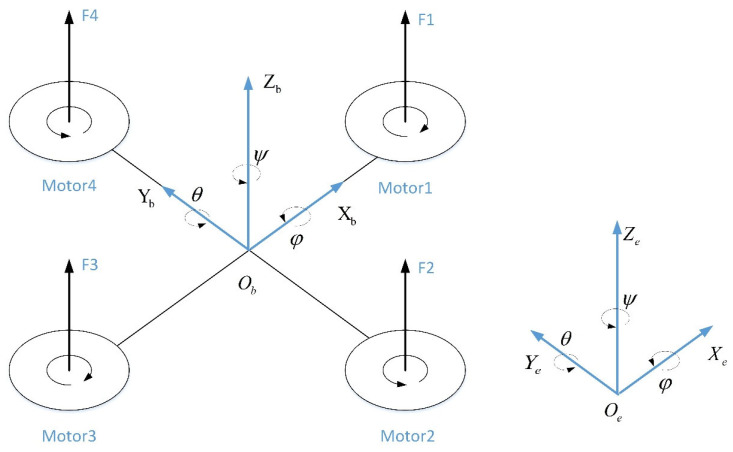
Diagram of quadrotor UAV structure.

The power system of the quadrotor drone is driven by four sets of rotor motors, with dynamic control of flight attitude and spatial position achieved by adjusting the speed of each motor. To accurately characterize the spatial state of quadrotor drones, the following two coordinate systems are defined, as shown in Figure 1:

Inertial frame $\left(X_{e},Y_{e},Z_{e}\right)$: Earth-fixed reference with $Z_{e}$ upward.

Body frame $\left(X_{b},Y_{b},Z_{b}\right)$: Origin at the quadrotor’s center of mass, with $X_{b}$ aligned with the heading direction.

The rotation matrix $R_{b}^{e}$ from the body frame to the inertial frame is

(1) $$R_{b}^{e}=\left[\begin{array}{ccc} c\;\psi \;c\;\theta & c\;\psi \;s\;\theta \;s\;\varphi -s\;\psi \;c\;\varphi & c\;\psi \;s\;\theta \;c\;\varphi +s\;\psi \;s\;\varphi \\ s\;\psi \;c\;\theta & s\;\psi \;s\;\theta \;s\;\varphi +c\;\psi \;c\;\varphi & s\;\psi \;s\;\theta \;c\;\varphi -s\;\psi \;s\;\varphi \\ -s\;\theta & c\;\theta \;s\;\varphi & c\;\theta \;c\;\varphi \end{array}\right]$$

where φ, θ,and ψ denote the roll, pitch, and yaw angles, and c θ and s θ denote cosθ and sinθ, respectively; this rule also applies to the parameters ψ and φ.

The translational and rotational dynamics are derived via the Newton–Euler method [26]:

(2) $$\left\{\begin{array}{c} ma=F_{D}+F_{G}+D_{p}\\ M_{L}+M_{F}+M_{T}=I\dot{\omega }+\omega \times \left(I\omega \right)+D_{q} \end{array}\right.$$

Here, m is the quadrotor mass, $a={\left[\ddot{x},\ddot{y},\ddot{z}\right]}^{T}$ represents the inertial acceleration of the drone, $I=diag\left(I_{x},I_{y},I_{z}\right)$ denotes the moment-of-inertia tensor, and $F_{G}$ is the gravity acting on the drone. $D_{p}=\left[d_{x},d_{y},d_{z}\right]$ represents the combined internal and external disturbances acting on the UAV’s position variables, where $d_{x}$, $d_{y}$, and $d_{z}$ denote disturbance along the x-, y-, and z-axes. Meanwhile, $D_{q}=\left[d_{\varphi },d_{\theta },d_{\psi }\right]$ represents the combined internal and external disturbances affecting the UAV’s attitude variables, where $d_{\varphi }$, $d_{\theta }$, and $d_{\psi }$ denote disturbance in the roll, pitch, and yaw angles. $M_{L}={\left[0,0,\gamma ({{-\omega }_{1}}^{2}+{\omega_{2}}^{2}-{\omega_{3}}^{2}+{\omega_{4}}^{2})\right]}^{T}$ denotes the sum of the lift moments generated by the rotor blades, $M_{F}={\left[l\rho ({\omega_{4}}^{2}-{\omega_{2}}^{2}),l\rho ({\omega_{3}}^{2}{{-\omega }_{1}}^{2}),0\right]}^{T}$ denotes the reaction torque produced by air resistance on the rotor [27], and $M_{T}={\left[I_{r}\dot{\theta }\omega_{r},-I_{r}\dot{\varphi }\omega_{r},0\right]}^{T}$ denotes the gyroscopic torque of the rotor. $F_{D}$ represents the total lift force generated by rotor rotation, expressed as

(3) $$F_{D}=R_{b}^{e}\left[\begin{array}{c} 0\\ 0\\ \rho \left({\omega_{1}}^{2}+{\omega_{2}}^{2}+{\omega_{3}}^{2}+{\omega_{4}}^{2}\right) \end{array}\right]$$

where ρ is the thrust coefficient, and $\omega_{j}\;\left(j=1,2,3,4\right)$ denotes the rotational speed of the j-th rotor motor.

The expression for the total moment of the quadrotor UAV is as follows:

(4) $$M_{L}+M_{F}+M_{T}=\left[\begin{array}{c} l\rho ({\omega_{4}}^{2}-{\omega_{2}}^{2})+I_{r}\dot{\theta }\omega_{r}\\ l\rho ({\omega_{3}}^{2}{{-\omega }_{1}}^{2})-I_{r}\dot{\varphi }\omega_{r}\\ \gamma ({{-\omega }_{1}}^{2}+{\omega_{2}}^{2}-{\omega_{3}}^{2}+{\omega_{4}}^{2}) \end{array}\right]$$

where l is the distance from the rotor to the UAV’s center of mass, and $I_{r}$ is the rotating propeller’s moment of inertia. γ is the rotational drag coefficient, characterizing the aerodynamic resistance during rotor rotation. $\omega_{r}$ represents the rotational angular velocity of the motor shaft, defined as $\omega_{r}=\omega_{1}-\omega_{2}+\omega_{3}-\omega_{4}$.

ω denotes the angular velocity vector of the UAV in the body frame. Under small-angle approximation (φ, θ ≈ 0), its relationship with attitude–angle derivatives is given by [28]

(5) $$\omega =\left[\begin{array}{ccc} 1 & 0 & -\sin\theta \\ 0 & \cos\varphi & \cos\theta \sin\varphi \\ 0 & -\sin\varphi & \cos\theta \cos\varphi \end{array}\right]\left[\begin{array}{c} \dot{\varphi }\\ \dot{\theta }\\ \dot{\psi } \end{array}\right]=\left[\begin{array}{c} \dot{\varphi }\\ \dot{\theta }\\ \dot{\psi } \end{array}\right]$$

Under small-angle assumptions, the 6-DoF dynamics simplify to

(6) $$\left\{\begin{array}{c} \ddot{x}=(sin\theta cos\varphi cos\psi +sin\varphi sin\psi )\frac{U_{1}}{m}+d_{x}\\ \ddot{y}=(sin\theta cos\varphi sin\psi -sin\varphi cos\psi )\frac{U_{1}}{m}+d_{y}\\ \ddot{z}=cos\varphi cos\theta \frac{U_{1}}{m}-g+d_{z}\\ \ddot{\varphi }=\frac{\left(I_{y}-I_{z}\right)}{I_{x}}\dot{\theta }\dot{\psi }+\frac{U_{2}}{I_{x}}+\frac{I_{r}}{I_{x}}\dot{\theta }\omega_{r}+d_{\varphi }\\ \ddot{\theta }=\frac{\left(I_{z}-I_{x}\right)}{I_{y}}\dot{\varphi }\dot{\psi }+\frac{U_{3}}{I_{y}}-\frac{I_{r}}{I_{y}}\dot{\varphi }\omega_{r}+d_{\theta }\\ \ddot{\psi }=\frac{\left(I_{x}-I_{y}\right)}{I_{z}}\dot{\varphi }\dot{\theta }+\frac{U_{4}}{I_{z}}+d_{\psi } \end{array}\right.$$

The control inputs $U_{1}$ (total thrust), $U_{2}$, $U_{3}$, and $U_{4}$ (roll/pitch/yaw moments) are as follows:

(7) $$\left\{\begin{array}{c} U_{1}=\rho ({\omega_{1}}^{2}+{\omega_{2}}^{2}+{\omega_{3}}^{2}+{\omega_{4}}^{2})\\ U_{2}=\rho l({\omega_{4}}^{2}-{\omega_{2}}^{2})\\ U_{3}=\rho l({\omega_{3}}^{2}{{-\omega }_{1}}^{2})\\ U_{4}=\gamma ({{-\omega }_{1}}^{2}+{\omega_{2}}^{2}-{\omega_{3}}^{2}+{\omega_{4}}^{2}) \end{array}\right.$$

### 2.2. Problem Formulation and Control Decoupling

Given the desired trajectory $\left(x^{d},y^{d},z^{d}\right)$ and yaw angle $\psi^{d}$, the quadrotor trajectory tracking problem is decoupled into position and attitude control. In position control, $\left(x,y,z\right)$ is adjusted through thrust $U_{1}$. In attitude control, $\psi^{d}$ is tracked while roll φ and pitch θ are treated as intermediate variables (rather than direct control targets). Due to the underactuated and strongly coupled nature of quadrotor dynamics, simultaneous control of all six degrees of freedom is infeasible. Thus, we design four control inputs $\left(U_{1},U_{2},U_{3},U_{4}\right)$ to stabilize $\left(x,y,z,\psi \right)$ at desired values, and we let φ and θ be implicitly regulated by position errors.

Define virtual control inputs $\left(u_{x},u_{y},u_{z}\right)$ for position error dynamics as follows:

(8) $$\left\{\begin{array}{c} u_{x}=(sin\theta cos\varphi cos\psi +sin\varphi sin\psi )\frac{U_{1}}{m}\\ u_{y}=(sin\theta cos\varphi sin\psi -sin\varphi cos\psi )\frac{U_{1}}{m}\\ u_{z}=cos\varphi cos\theta \frac{U_{1}}{m}-g \end{array}\right.$$

Based on (8), solve for total thrust $U_{1}$ and the desired roll/pitch angles $\left(\varphi^{d},\theta^{d}\right)$:

(9) $$\left\{\begin{array}{l} U_{1}=m\sqrt{{u_{x}}^{2}+{u_{y}}^{2}+{\left(u_{z}+g\right)}^{2}}\\ \varphi^{d}=arcsin\left(m\frac{u_{y}cos\psi -u_{x}sin\psi }{U_{1}}\right)\\ \theta^{d}=arctan\left(\frac{u_{x}cos\psi +u_{y}sin\psi }{u_{z}+g}\right) \end{array}\right.$$

**Figure 2:**
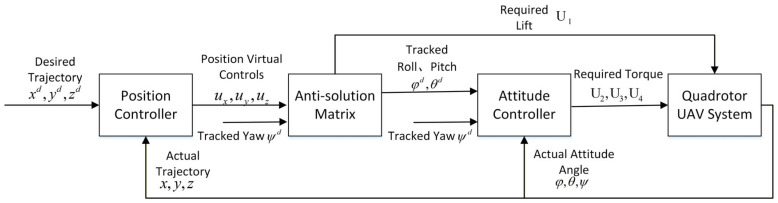
Control block diagram of quadcopter UAV.

The attitude controller computes $\left(U_{2},U_{3},U_{4}\right)$ using errors in $\left(\varphi^{d},\theta^{d},\psi^{d}\right)$ to achieve roll/pitch tracking for position stabilization and direct yaw control for orientation. The four control inputs $\left(U_{1},U_{2},U_{3},U_{4}\right)$ are mapped to rotor speeds $\left(\omega_{1},\omega_{2},\omega_{3},\omega_{4}\right)$ via (7). The overall control architecture of the quadrotor UAV adopts a dual-loop control scheme, as shown in Figure 2. It consists of an outer (position) loop that generates $\left(\varphi^{d},\theta^{d},U_{1}\right)$ from trajectory errors and an inner (attitude) loop that computes $\left(U_{2},U_{3},U_{4}\right)$ for attitude tracking.

## 3. Controller Design and Stability Analysis

### 3.1. Position-Loop Controller Design

Given the identical design principles for x-, y-, and z-axis position controllers, we represent the design using z-axis dynamics by combining (6) and (8) as follows:

(10) $$\ddot{z}=cos\varphi cos\theta \frac{U_{1}}{m}-g+d_{z}=u_{z}+d_{z}$$

The following global fast terminal sliding mode surface is proposed:

(11) $$s_{z}=\dot{e_{z}}+\alpha {\left|e_{z}\right|}^{\gamma }sgn\left(e_{z}\right)+\beta {\left|e_{z}\right|}^{\rho }sgn\left(e_{z}\right)$$

where $e_{z}=z-z^{d}$ denotes the position tracking error. α,β>0, γ>1, and 0<ρ<1.

To enhance convergence dynamics, the following dual power reaching law is introduced:

(12) $$\dot{s_{z}}=-k_{1}{\left|s_{z}\right|}^{\gamma^{\prime }}sgn\left(s_{z}\right)-k_{2}{\left|s_{z}\right|}^{\rho^{\prime }}sgn\left(s_{z}\right)-k_{0}sgn\left(s_{z}\right)+d_{z}$$

where $k_{i}\;\left(i=0,1,2\right)>0$, $\gamma^{\prime }>1$, ${0<\rho }^{\prime }<1$, and $\left|d_{z}\right|\leq D_{z}$, with $D_{z}$ being the disturbance bound.

In the operation of the control system, the double power approximation law realizes dynamic optimization through its unique dual-modal adjustment mechanism. When there is a large deviation between the system state and the sliding mode surface, the high-power term (index greater than one) is used as the dominant factor to accelerate the convergence of the system to the sliding mode surface. As the sliding surface is approached, the low power term (exponent less than one) becomes the dominant factor, serving the same function. The two items in the double power approach law work synergistically in the process of system stabilization, so as to effectively improve the dynamic characteristics of the approach process and significantly enhance the stability performance of the system in the global range.

By differentiating (11), we obtain

(13) $$\begin{array}{c} \dot{s_{z}}=\ddot{e_{z}}+\alpha \gamma {\left|e_{z}\right|}^{\gamma -1}sgn\left(e_{z}\right)+\beta \rho {\left|e_{z}\right|}^{\rho -1}sgn\left(e_{z}\right) \end{array}\;\begin{array}{c} =\ddot{z}-\ddot{z^{d}}+\alpha \gamma {\left|e_{z}\right|}^{\gamma -1}sgn\left(e_{z}\right)+\beta \rho {\left|e_{z}\right|}^{\rho -1}sgn\left(e_{z}\right)\; \end{array}$$

The z-axis controller is designed as follows based on Equations (12) and (13):

(14) $$\;u_{z}=\ddot{z^{d}}-\alpha \gamma {\left|e_{z}\right|}^{\gamma -1}sgn\left(e_{z}\right)-\beta \rho {\left|sat\left(e_{z},d\right)\right|}^{\rho -1}sgn\left(e_{z}\right)-\;k_{1}{\left|s_{z}\right|}^{\gamma^{\prime }}sgn\left(s_{z}\right)-k_{2}{\left|s_{z}\right|}^{\rho^{\prime }}sgn\left(s_{z}\right)-k_{0}sgn\left(s_{z}\right)$$

To avoid singularity issues (caused by ρ−1<0), the following saturation function is applied:

(15) $$sat\left(e_{z},d\right)=\left\{\begin{array}{cc} e_{z} & e_{z}\geq d\\ d*sgn\left(e_{z}\right) & e_{z}<d \end{array}\right.$$

where d is the saturation threshold and d>0.

The full position-loop controller for the (x, y, z) axes is

(16) $$\left\{\begin{array}{c} \begin{array}{c} u_{x}={\ddot{x}}^{d}-\alpha_{x}\gamma_{x}{\left|e_{x}\right|}^{\gamma_{x}-1}\mathrm{sgn}\left(e_{x}\right)-\beta_{x}\rho_{x}{\left|sat\left(e_{x},d\right)\right|}^{\rho_{x}-1}\mathrm{sgn}\left(e_{x}\right)-\\ k_{x1}{\left|s_{x}\right|}^{\gamma_{x}^{\prime }}\mathrm{sgn}\left(s_{x}\right)-k_{x2}{\left|s_{x}\right|}^{\rho_{x}^{\prime }}\mathrm{sgn}\left(s_{x}\right)-k_{x0}\mathrm{sgn}\left(s_{x}\right)\;\;\; \end{array}\\ \begin{array}{c} u_{y}={\ddot{y}}^{d}-\alpha_{y}\gamma_{y}{\left|e_{y}\right|}^{\gamma_{y}-1}\mathrm{sgn}\left(e_{y}\right)-\beta_{y}\rho_{y}{\left|sat\left(e_{y},d\right)\right|}^{\rho_{y}-1}\mathrm{sgn}\left(e_{y}\right)-\\ k_{y1}{\left|s_{y}\right|}^{\gamma_{y}^{\prime }}\mathrm{sgn}\left(s_{y}\right)-k_{iy2}{\left|s_{y}\right|}^{\rho_{y}^{\prime }}\mathrm{sgn}\left(s_{y}\right)-k_{y0}\mathrm{sgn}\left(s_{y}\right)\;\;\; \end{array}\\ \begin{array}{c} u_{z}={\ddot{z}}^{d}-\alpha_{z}\gamma_{z}{\left|e_{z}\right|}^{\gamma_{z}-1}\mathrm{sgn}\left(e_{z}\right)-\beta_{z}\rho_{z}{\left|sat\left(e_{z},d\right)\right|}^{\rho_{z}-1}\mathrm{sgn}\left(e_{z}\right)-\\ k_{z1}{\left|s_{z}\right|}^{\gamma_{z}^{\prime }}\mathrm{sgn}\left(s_{z}\right)-k_{z2}{\left|s_{z}\right|}^{\rho_{z}^{\prime }}\mathrm{sgn}\left(s_{z}\right)-k_{z0}\mathrm{sgn}\left(s_{z}\right)\;\;\; \end{array} \end{array}\right.$$

where $e_{x}=x-x^{d}$, $e_{y}=y-y^{d}$, and $e_{z}=z-z^{d}$ represent position tracking errors along the x-, y-, and z-axes, respectively. $s_{x}$, $s_{y}$, and $s_{z}$ denote the sliding surfaces for the three axes. $\gamma_{x},\gamma_{x}^{\prime }>1$, $\gamma_{y},\gamma_{y}^{\prime }>1$, and $\gamma_{z},\gamma_{z}^{\prime }>1$ are high power exponents for fast convergence, while $0<\rho_{x},\rho_{x}^{\prime }<1$, $0<\rho_{y},\rho_{y}^{\prime }<1$, and $0<\rho_{z},\rho_{z}^{\prime }<1$ are low power exponents for smooth stabilization. $\alpha_{x}$, $\alpha_{y}$, $\alpha_{z}$, $\beta_{x}$, $\beta_{y}$, $\beta_{z}$, $k_{xi}\;\left(i=0,1,2\right)$, $k_{yi}\;\left(i=0,1,2\right)$, and $k_{zi\;}\left(i=0,1,2\right)$ are all positive.

### 3.2. Stability Analysis

Given the symmetric structure of the position-loop controller (16), the stability of all three channels (x, y, z) can be inferred by analyzing any single channel. We select the z-axis for demonstration.

**Theorem** **1.** *Consider the second-order z-axis dynamics of the quadrotor UAV (6) with bounded disturbance $\left|d_{z}\right|\leq D$. If the control input is designed according to the terminal sliding mode control law (12) based on the sliding surface (11), then the sliding variable $s_{z}$ will reach and remain on the sliding surface $s_{z}=0$ within finite time $t_{r}$, the tracking error $e_{z}$ will converge to zero within $t_{r}+t_{s}$, and the system is globally finite-time stable*.

**Proof of Theorem** **1.** The proof consists of two phases, as shown below.

Phase 1: Finite-time reachability ($s_{z}\to 0$).

Consider the Lyapunov function candidate

(17) $$V_{z}=\frac{1}{2}{s_{z}}^{2}$$

Differentiating $V_{z}$ yields

(18) $$\dot{V_{z}}=s_{z}\left(-k_{1}{\left|s_{z}\right|}^{\gamma^{\prime }}sgn\left(s_{z}\right)-k_{2}{\left|s_{z}\right|}^{\rho^{\prime }}sgn\left(s_{z}\right)-k_{0}sgn\left(s_{z}\right){+d}_{z}\right)\;=-k_{1}{\left|s_{z}\right|}^{1+\gamma^{\prime }}-k_{2}{\left|s_{z}\right|}^{1+\rho^{\prime }}-k_{0}\left|s_{z}\right|+sd_{z}$$

Given $\left|d_{z}\right|\leq D_{z}$ and $sd_{z}\leq \left|s_{z}\right|D_{z}$,

(19) $$\dot{V_{z}}\leq -k_{1}{\left|s_{z}\right|}^{1+\gamma^{\prime }}-k_{2}{\left|s_{z}\right|}^{1+\rho^{\prime }}-(k_{0}-D_{z})\left|s_{z}\right|$$

Selecting $k_{0}\geq D_{z}$ yields

(20) $$\dot{V_{z}}\leq -k_{1}{\left|s_{z}\right|}^{1+\gamma^{\prime }}-k_{2}{\left|s_{z}\right|}^{1+\rho^{\prime }}\leq -k_{1}2^{\frac{1+\gamma^{\prime }}{2}}{\left(V_{z}\right)}^{\frac{1+\gamma^{\prime }}{2}}-k_{2}2^{\frac{1+\rho^{\prime }}{2}}{\left(V_{z}\right)}^{\frac{1+\rho^{\prime }}{2}}$$

This satisfies the conditions of Lemma 1 [29], guaranteeing finite-time convergence to $s_{z}=0$ within

(21) $$t_{r}<\frac{1}{a_{1}(b_{1}-1)}+\frac{1}{a_{2}(1-b_{2})}$$

where $a_{1}=k_{1}2^{\frac{1+\gamma^{\prime }}{2}}$, $a_{2}=k_{2}2^{\frac{1+\rho^{\prime }}{2}}$, $b_{1}=\frac{1+\gamma^{\prime }}{2}$, and $b_{2}=\frac{1+\rho^{\prime }}{2}$.

Phase 2: Finite-time error convergence ($e_{z}\to 0$).

When $s_{z}=0$, the system dynamics reduce to $\dot{e_{z}}=-\alpha {\left|e_{z}\right|}^{\gamma }sgn\left(e_{z}\right)-\beta {\left|e_{z}\right|}^{\rho }sgn\left(e_{z}\right)$. Through Lemma 2 [30], $e_{z}$ converges to zero in finite time: $t_{s}<\frac{1}{\alpha (\gamma -1)}+\frac{1}{\beta (1-\rho )}$.

Conclusion: The tracking error $e_{z}$ converges to zero within $t_{r}$ + $t_{s}$, proving global finite-time stability. □

**Lemma 1**  ([29])**.** *For a second-order system, if there exists a Lyapunov function satisfying*

(22) $$\dot{V}\left(x\right)\leq -aV{\left(x\right)}^{\alpha }-bV{\left(x\right)}^{\beta }$$

*where a,b>0 and 0<β<1<α, then the system is globally fixed-time stable with settling time*

(23) $$T\leq \frac{1}{a(\alpha -1)}+\frac{1}{b(1-\beta )}$$

**Lemma 2**  ([30])**.** *For a second-order system with sliding surface*

(24) $$s=y_{2}+\alpha {\left|y_{1}\right|}^{\gamma }sgn\left(y_{1}\right)+\beta {\left|y_{1}\right|}^{\rho }sgn\left(y_{1}\right)$$

*where γ>1 and 0<ρ<1, the equilibrium point is globally asymptotically stable with convergence time*

(25) $$T<\frac{1}{\alpha (\gamma -1)}+\frac{1}{\beta (1-\rho )}$$

### 3.3. Attitude-Loop Controller Design

The design principles for all three attitude controllers (φ, θ, ψ) are identical. We demonstrate the design using the roll angle φ as an example.

The roll-angle dynamics are given by

(26) $$\ddot{\varphi }=\frac{U_{2}}{I_{x}}+f(\dot{\theta },\dot{\psi })+d_{\varphi }$$

where

(27) $$f(\dot{\theta },\dot{\psi })=\frac{\left(I_{y}-I_{z}\right)}{I_{x}}\dot{\theta }\dot{\psi }+\frac{I_{r}}{I_{x}}\dot{\theta }\omega_{r}$$

The sliding surface can be formulated as

(28) $$s_{\varphi }=\dot{e_{\varphi }}+\alpha {\left|e_{\varphi }\right|}^{\gamma }sgn\left(e_{\varphi }\right)+\beta {\left|e_{\varphi }\right|}^{\rho }sgn\left(e_{\varphi }\right)$$

where $e_{\varphi }=\varphi -\varphi^{d}$, α,β>0, γ>1, 0<ρ<1.

The dual power reaching law can be expressed as

(29) $$\dot{s_{\varphi }}=-k_{1}{\left|s_{\varphi }\right|}^{\gamma^{\prime }}sgn\left(s_{z}\right)-k_{2}{\left|s_{\varphi }\right|}^{\rho^{\prime }}sgn\left(s_{z}\right)-k_{0}sgn\left(s_{\varphi }\right)+d_{\varphi }$$

where $k_{i\;}\left(i=0,1,2\right)>0$, $\gamma^{\prime }>1$, ${0<\rho }^{\prime }<1$, and $\left|d_{z}\right|\leq D_{\varphi }$, with $D_{\varphi }$ being the disturbance bound.

The roll-angle controller is formulated as

(30) $$U_{2}=I_{x}({\ddot{\varphi }}^{d}-f\left(\dot{\theta },\dot{\psi }\right)-\alpha \gamma {\left|e_{\varphi }\right|}^{\gamma -1}sgn\left(e_{\varphi }\right)-\beta \rho {\left|sat\left(e_{\varphi },d\right)\right|}^{\rho -1}sgn\left(e_{\varphi }\right)-\;k_{1}{\left|s_{\varphi }\right|}^{\gamma^{\prime }}sgn\left(s_{\varphi }\right)-k_{2}{\left|s_{\varphi }\right|}^{\rho^{\prime }}sgn\left(s_{\varphi }\right)-k_{0}sgn\left(s_{\varphi }\right))$$

where $k_{i}\;\left(i=0,1,2\right)>0$, $\gamma^{\prime }>1$, and ${0<\rho }^{\prime }<1$.

Based on the roll-angle controller derivation, the complete attitude-loop controller for (φ, θ, ψ) can be expressed as

(31) $$\left\{\begin{array}{c} \begin{array}{c} U_{2}=I_{x}({\ddot{\varphi }}^{d}-f\left(\dot{\theta },\dot{\psi }\right)-\alpha_{\varphi }\gamma_{\varphi }{\left|e_{\varphi }\right|}^{\gamma_{\varphi }-1}\mathrm{sgn}\left(e_{\varphi }\right)-\beta_{\varphi }\rho_{\varphi }{\left|sat\left(e_{\varphi },d\right)\right|}^{\rho_{\varphi }-1}\mathrm{sgn}\left(e_{\varphi }\right)-\\ k_{\varphi 1}{\left|s_{\varphi }\right|}^{\gamma_{\varphi }^{\prime }}\mathrm{sgn}\left(s_{\varphi }\right)-k_{\varphi 2}{\left|s_{\varphi }\right|}^{\rho_{\varphi }^{\prime }}\mathrm{sgn}\left(s_{\varphi }\right)-k_{\varphi 0}\mathrm{sgn}\left(s_{\varphi }\right))\;\;\;\;\;\; \end{array}\\ \begin{array}{c} U_{3}=I_{y}({\ddot{\theta }}^{d}-f\left(\dot{\varphi },\dot{\psi }\right)-\alpha_{\theta }\gamma_{\theta }{\left|e_{\theta }\right|}^{\gamma_{\varphi }-1}\mathrm{sgn}\left(e_{\theta }\right)-\beta_{\theta }\rho_{\theta }{\left|sat\left(e_{\theta },d\right)\right|}^{\rho_{\varphi }-1}\mathrm{sgn}\left(e_{\theta }\right)-\\ k_{\theta 1}{\left|s_{\theta }\right|}^{\gamma_{\theta }^{\prime }}\mathrm{sgn}\left(s_{\theta }\right)-k_{\theta 2}{\left|s_{\theta }\right|}^{\rho_{\theta }^{\prime }}\mathrm{sgn}\left(s_{\theta }\right)-k_{\theta 0}\mathrm{sgn}\left(s_{\theta }\right))\;\;\;\;\;\;\; \end{array}\\ \begin{array}{c} U_{4}=I_{z}({\ddot{\Psi }}^{d}-f\left(\dot{\varphi },\dot{\theta }\right)-\alpha_{\Psi }\gamma_{\Psi }{\left|e_{\Psi }\right|}^{\gamma_{\Psi }-1}\mathrm{sgn}\left(e_{\Psi }\right)-\beta_{\Psi }\rho_{\Psi }{\left|sat\left(e_{\Psi },d\right)\right|}^{\rho_{\Psi }-1}\mathrm{sgn}\left(e_{\Psi }\right)-\\ k_{\Psi 1}{\left|s_{\Psi }\right|}^{\gamma_{\Psi }^{\prime }}\mathrm{sgn}\left(s_{\Psi }\right)-k_{\Psi 2}{\left|s_{\Psi }\right|}^{\rho_{\Psi }^{\prime }}\mathrm{sgn}\left(s_{\Psi }\right)-k_{\Psi 0}\mathrm{sgn}\left(s_{\Psi }\right))\;\;\;\;\;\; \end{array} \end{array}\right.$$

where $e_{\varphi }=\varphi -\varphi^{d}$, $e_{\theta }=\theta -\theta^{d}$, and $e_{\psi }=\psi -\psi^{d}$ represent attitude tracking errors. $s_{\varphi }$, $s_{\theta }$, and $s_{\psi }$ denote the sliding surfaces for three attitude angles. $\gamma_{\varphi },\gamma_{\varphi }^{\prime }>1$, $\gamma_{\theta },\gamma_{\theta }^{\prime }>1$, and $\gamma_{\psi },\gamma_{\psi }^{\prime }>1$ are high power exponents for fast convergence, while $0<\rho_{\varphi },\rho_{\varphi }^{\prime }<1$, $0<\rho_{\theta },\rho_{\theta }^{\prime }<1$, and $0<\rho_{\psi },\rho_{\psi }^{\prime }<1$ are low power exponents for smooth stabilization. $\alpha_{\varphi }$, $\alpha_{\theta }$, $\alpha_{\psi }$, $\beta_{\varphi }$, $\beta_{\theta }$, $\beta_{\psi }$, $k_{\varphi i}\;\left(i=0,1,2\right)$, $k_{\theta i}\;\left(i=0,1,2\right)$, $k_{\psi i}\;\left(i=0,1,2\right)$ are all positive.

It can be seen that the tracking-error dynamics and sliding surface, reaching law, and controller design of both the position loop and attitude loop are consistent in form. Consequently, the stability proof for the attitude loop is identical to the analysis process of the position loop. Theorem 1 is also applicable to the attitude loop, and the designed nonsingular terminal sliding mode controller for the attitude loop can guarantee that the tracking error of the attitude-angle states in the system’s attitude loop converges to zero within finite time.

### 3.4. Stability Analysis of Controllers Under Saturated Functions

To reduce the chattering effect caused by the sign function $sgn\left(s\right)$ in the control law and ensure control continuity, all three sliding mode methods (GFTSMC, NTSMC, SMC) employ a saturation function sat(s) instead of the sign function $sgn\left(s\right)$ in Equations (14) and (29) as follows:

$$sat(s)=\left\{\begin{array}{c} 1,s>∆\\ \frac{s}{∆},\left|s\right|\leq ∆\\ -1,s<-∆ \end{array}\right.$$

where ∆>0 represents the boundary layer thickness set to ∆=0.15 for all controllers to ensure fair comparison.

From the structural characteristics of the position loop and attitude loop controllers, it can be concluded that the controllers have the same form. Therefore, it is only necessary to prove the stability of any single channel, and the stability of the remaining channels can be inferred by analogy. The following takes the z-axis as an example to prove the stability of the controller under saturated functions.

The design of the z-axis controller under saturated functions is as follows:

(32) $$u_{z}=\ddot{z^{d}}-\alpha \gamma {\left|e_{z}\right|}^{\gamma -1}sgn\left(e_{z}\right)-\beta \rho {\left|sat\left(e_{z},d\right)\right|}^{\rho -1}sgn\left(e_{z}\right)-\;\;\;\;\;\;k_{1}{\left|s_{z}\right|}^{\gamma^{\prime }}sgn\left(s_{z}\right)-k_{2}{\left|s_{z}\right|}^{\rho^{\prime }}sgn\left(s_{z}\right)-k_{0}sat\left(s_{z}\right)$$

The proof is divided into two parts. In the first part, it is first proved that the sliding mode variable $s_{z}$ converges to the boundary layer $\left|s\right|\leq ∆$ in finite time. In the second part, it is proved that the sliding mode variable $s_{z}$ and the error $e_{z}$ are uniformly ultimately bounded within the boundary layer. The first part has been proved in the previous text, and the proof of the uniform ultimate boundedness of variables in the second part is presented herein.

**Theorem** **2** (Uniform Ultimate Boundedness [31])**.** *For the variable $x\left(t\right)$, if there exists a bounded region radius δ independent of the initial time $t_{0}$, and a convergence time T related only to δ and the bound of initial states, such that $\left|x\left(t\right)\right|\leq \delta$ when $t\geq t_{0}+T$, then the state $x\left(t\right)$ is uniformly ultimately bounded.*

Phase 1: $s_{z}$ is uniformly ultimately bounded.

From $\left|s\right|\leq ∆$ and combined with Equation (12), it can be derived that

(33) $$\left|\dot{s_{z}}\right|\leq k_{1}{∆}^{\gamma^{\prime }}+k_{2}{∆}^{\rho^{\prime }}+k_{0}+D=M$$

where $M=k_{1}{∆}^{\gamma^{\prime }}+\;k_{2}{∆}^{\rho^{\prime }}+\;k_{0}+\;D$ is a constant. It can thus be concluded that there exists $t\geq t_{1}$ such that $\left|s\left(t\right)\right|\leq \;\delta_{s}$. Consequently, the sliding mode variable $s_{z}$ satisfies the property of ultimate uniform boundedness.

Phase 2: $e_{z}$ is uniformly ultimately bounded.

Let $\alpha {\left|e_{z0}\right|}^{\gamma }sgn\left(e_{z0}\right)+\beta {\left|e_{z0}\right|}^{\rho }sgn\left(e_{z0}\right)=∆$.

When the error $e_{z}$ is large (i.e., $\left|e_{z}\right|\geq e_{z0}$), $\alpha {\left|e_{z}\right|}^{\gamma }sgn\left(e_{z}\right)+\beta {\left|e_{z}\right|}^{\rho }sgn\left(e_{z}\right)>∆$.

If $e_{z}>0$, it follows from the equation that $\dot{e_{z}}\leq ∆-\alpha {\left|e_{z}\right|}^{\gamma }-\beta {\left|e_{z}\right|}^{\rho }<0$. If $e_{z}<0$, then $\dot{e_{z}}\geq -∆+\alpha {\left|e_{z}\right|}^{\gamma }+\beta {\left|e_{z}\right|}^{\rho }>0$.

It can thus be concluded that when the error $e_{z}$ is large, $e_{z}\cdot \dot{e_{z}}<0$; this implies the error $e_{z}$ converges toward the origin and satisfies the property of ultimate uniform boundedness.

When the error $e_{z}$ is small (i.e., $\left|e_{z}\right|\leq e_{z0}$), it follows from $\left|s\right|\leq ∆$ that $\left|\dot{e_{z}}\right|\leq ∆+\alpha {\left|e_{z0}\right|}^{\gamma }sgn\left(e_{z0}\right)+\beta {\left|e_{z0}\right|}^{\rho }sgn\left(e_{z0}\right)$. Let $M=∆+\alpha {\left|e_{z0}\right|}^{\gamma }sgn\left(e_{z0}\right)+\beta {\left|e_{z0}\right|}^{\rho }sgn\left(e_{z0}\right)$. Since M is independent of $e_{z}$, it can be concluded that there exists a time instant $t\geq t_{1}+t_{2}$ such that $\left|e_{z}\right|\leq B$, which implies $e_{z}$ satisfies the property of ultimate uniform boundedness.

In summary, there exists a constant B>0 such that $\left|e_{z}\right|\leq \delta_{e}$ when $t\geq t_{1}+t_{2}$.

In conclusion, the designed nonsingular terminal sliding mode controller under saturated functions can ensure that the tracking error of the system state satisfies the property of ultimate uniform boundedness.

## 4. Simulation and Analysis

### 4.1. Simulation Setup

To verify the effectiveness of the proposed GFTSMC algorithm, we conducted comprehensive simulations based on the quadrotor UAV model using MATLAB 2020b/Simulink. The structural parameters of the quadrotor UAV system are listed in Table 1.

The initial position and attitude of the quadrotor are set to 0, with the reference trajectory being a spiral curve as follows: $x^{d}=0.5sin(0.5t)$; $y^{d}=0.7cos(0.5t)$; and $z^{d}=0.5t$. The reference heading angle is $\psi^{d}=1$.

To simulate the effects of natural wind disturbances during flight, the wind disturbances model is constructed as a composite of two components: steady-state wind disturbance and gradually varying wind disturbance. Steady-state wind disturbance is modeled as a time-invariant constant, with its magnitude set proportional to the UAV mass to ensure that wind-load effects remain gravitationally significant. This scaling ensures that aerodynamic disturbances are physically meaningful relative to gravity. Gradually varying wind disturbance is modeled as band-limited white noise with a mean amplitude of 0.2, sampling frequency of 0.01 s, and a single-sided power spectral density of $4\times 10^{-4}$ to represent disturbances caused by random wind gusts and atmospheric turbulence.

The combined wind disturbance is configured such that the wind disturbance remains at 0 for the initial 5 s, while the disturbance conditions subsequent to this period are defined as follows:

$$\left\{\begin{array}{l} d_{x}=m+d_{b}\\ d_{y}=2m+d_{b}\\ d_{z}=0.5m+d_{b} \end{array}\right.$$

where $\left[d_{x},d_{y},d_{z}\right]$ denote disturbance along the x-, y-, and z-axes. $\left[m,2m,0.5m\right]$ represent the mass-related fixed disturbances corresponding to the three axes (where m denotes the mass of the UAV). $d_{b}$ denotes the gradually varying wind disturbance configured as a white noise signal, with a sampling time $T\left(d_{b}\right)=0.01\;s$, a sampling frequency $f\left(d_{b}\right)=100$, and a single-sided power spectral density $S\left(d_{b}\right)=4\times 10^{-4}$.

The design of the proposed GFTSMC algorithm’s parameters is outlined below.

Position-loop controller parameters:

$\gamma_{x}=\gamma_{y}=\gamma_{z}$ = 1.4; $\rho_{x}=\rho_{y}=\rho_{z}=\frac{1}{3}$;

$\gamma_{x}^{\prime }=\gamma_{y}^{\prime }=\gamma_{z}^{\prime }$ = 1.4; $\rho_{x}^{\prime }=\rho_{y}^{\prime }=\rho_{z}^{\prime }=\frac{1}{3}$;

$\alpha_{x}=1.5$, $\alpha_{y}=1.8$, $\alpha_{z}=1.1$; $\beta_{x}=1.5$, $\beta_{y}=1.8$, $\beta_{z}=1.1$;

$k_{x1}=k_{y1}=k_{z1}=10$; $k_{x2}=k_{y2}=k_{z2}=1.3$;

$k_{x0}=1.5$, $k_{y0}=2$, $k_{z0}=3$

Attitude-loop controller parameters:

$\gamma_{\varphi }=\gamma_{\theta }=\gamma_{\psi }$ = 1.4; $\rho_{\varphi }=\rho_{\theta }=\rho_{\psi }=\frac{1}{3}$;

$\gamma_{\varphi }^{\prime }=\gamma_{\theta }^{\prime }=\gamma_{\psi }^{\prime }$ = 1.4; $\rho_{\varphi }^{\prime }=\rho_{\theta }^{\prime }=\rho_{\psi }^{\prime }=\frac{1}{3}$;

$\alpha_{\varphi }=60$, $\alpha_{\theta }=60$, $\alpha_{\psi }=30$; $\beta_{\varphi }=60$, $\beta_{\theta }=60$, $\beta_{\psi }=30$;

$k_{\varphi 1}=k_{\theta 1}=k_{\psi 1}=100$; $k_{\varphi 2}=k_{\theta 2}=k_{\psi 2}=20$;

$k_{\varphi 0}=2.2$, $k_{\theta 0}=2.2$, $k_{\psi 0}=2.2$.

To validate the superiority of the proposed algorithm, we conduct simulations comparing GFTSMC to PID control, conventional SMC, and NTSMC.

### 4.2. Analysis of Simulation Results

**Figure 3:**
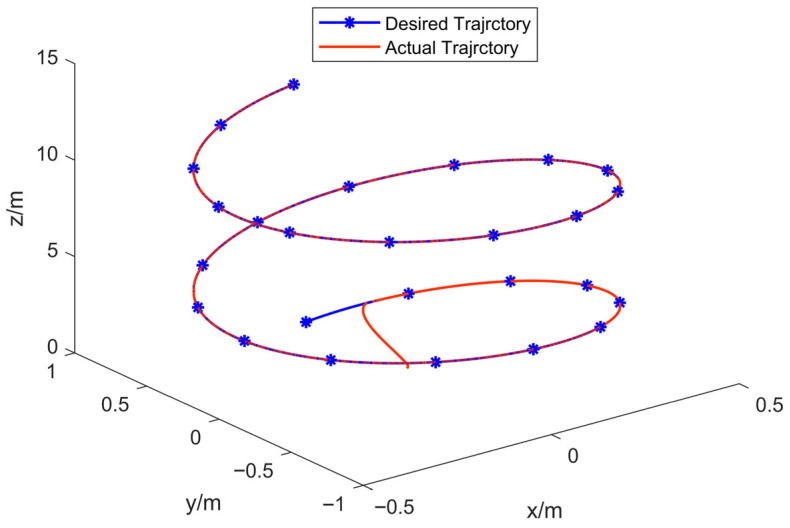
Curves of three-dimensional trajectory.

In Figure 3, the curves of three-dimensional trajectory present the 3D trajectory tracking performance of the quadrotor drone under GFTSMC. The results demonstrate that the actual trajectory precisely coincides with the desired trajectory once the UAV enters the prescribed flight path, indicating excellent tracking stability and control accuracy.

**Figure 4:**
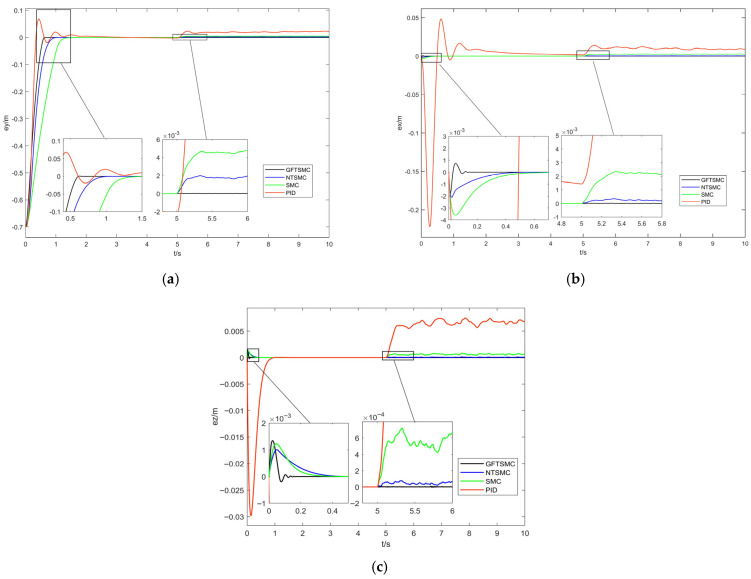
X, Y, and Z directional views of position-loop tracking errors under four control strategies. (**a**) X-direction view. (**b**) Y-direction view. (**c**) Z-direction view.

Figure 4 presents the three-axis tracking-error response curves of the quadrotor UAV under the four control methods. Table 2 presents the steady-state convergence time of the four control methods for reaching the desired three-axis positions. Table 3 presents the maximum tracking error after introducing disturbances of the four control methods. The simulation results show the following:

1. Tracking-Error Convergence Speed: The steady-state convergence time of GFTSMC for reaching the desired position on the x-axis is 0.13 s. Compared to NTSMC, SMC, and PID control, its steady-state convergence speed is increased by 72.3%, 78.3%, and 95.0%, respectively. For the desired y-axis position, the steady-state convergence time of GFTSMC is 0.64 s; in comparison with NTSMC, SMC, and PID control, this represents a 36%, 54.3%, and 70.0% improvement in steady-state convergence speed, respectively. Regarding the desired z-axis position, GFTSMC has a steady-state convergence time of 0.12 s, with its steady-state convergence speed increased by 60%, 67.5%, and 87.0% compared to NTSMC, SMC, and PID control, respectively. These comparative simulation results indicate that GFTSMC can effectively improve the convergence speed of the quadrotor UAV’s position loop.
2. Anti-Disturbance Performance: When a disturbance is introduced between 5 and 10 s, GFTSMC exhibits the smallest disturbance error, followed by NTSMC and SMC, while PID control shows the largest disturbance error. Compared to PID control, GFTSMC achieves a significant reduction in steady-state disturbance error, also demonstrating values reduced by 97.3% and 83.1% relative to SMC and NTSMC, respectively. The comparative simulation results demonstrate that GFTSMC can effectively mitigate the impact of external disturbances on system stability and enhance the anti-disturbance capability of the quadrotor UAV’s position loop.

**Figure 5:**
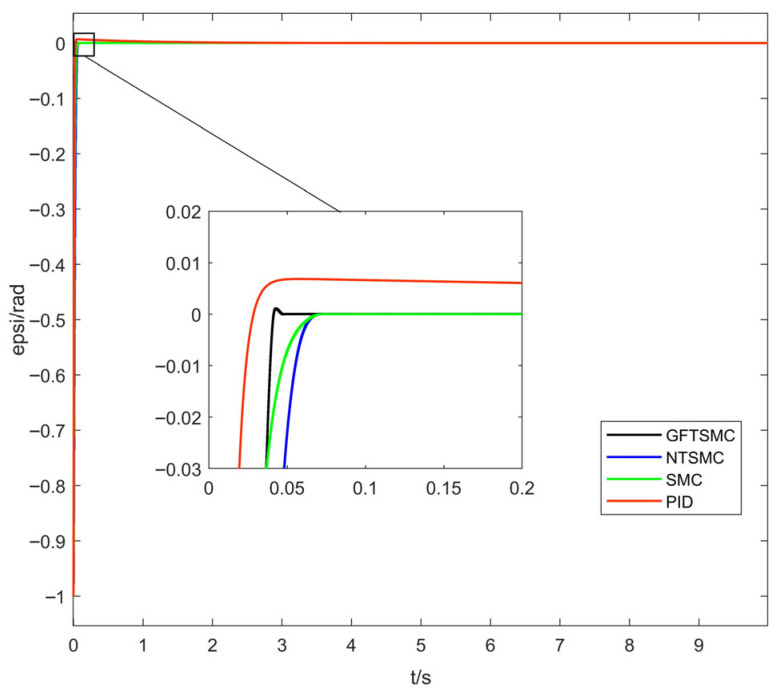
Response curves of yaw-angle tracking error.

Figure 5 shows the yaw-angle tracking-error response curves of the quadrotor UAV under the four control methods. Table 2 shows the steady-state convergence time of the four control methods for reaching the desired yaw angle. The simulation results indicate that GFTSMC’s convergence time to reach the desired yaw angle is 0.047 s, which is 0.024 s, 0.027 s, and 2 s less than that of NTSMC, SMC, and PID control, respectively. Compared to PID control, SMC, and NTSMC, the steady-state convergence time of GFTSMC is reduced by 97.7%, 36.5%, and 33.8%, respectively. Therefore, GFTSMC can effectively improve the convergence speed of the attitude loop of the quadrotor UAV.

**Figure 6:**
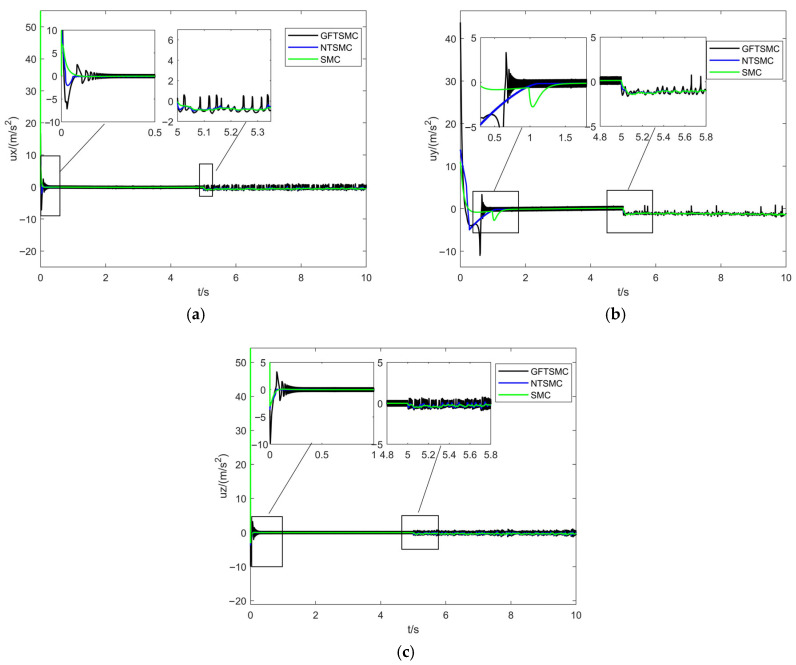
X, Y, and Z directional views of position-loop virtual control quantities under three control strategies. (**a**) $u_{x}$. (**b**) $u_{x}$. (**c**) $u_{x}$.

**Figure 7:**
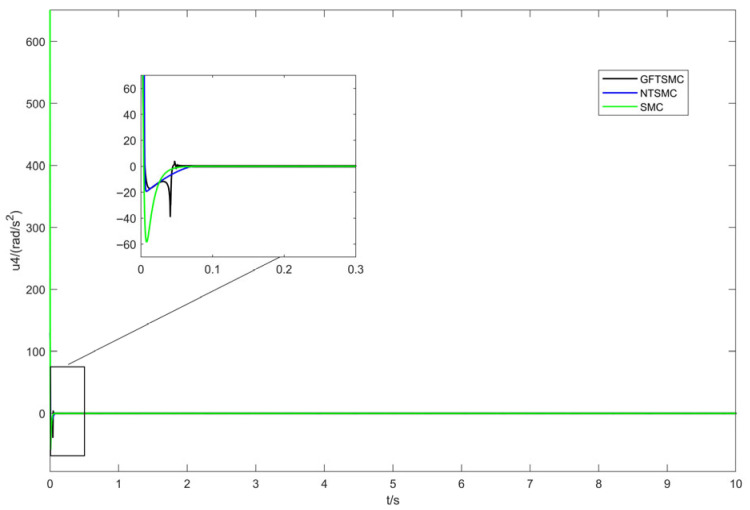
Response curves of yaw-angle control quantity.

Figure 6 and Figure 7 show the virtual control inputs and yaw moment responses. While discontinuity of the control input is an unavoidable issue in sliding mode control, the simulation results show the following:

1. Control Continuity: Using the saturation function effectively eliminates chattering while maintaining control continuity in GFTSMC.
2. Control Magnitude: The control input amplitude of GFTSMC is similar to that of NTSMC and SMC. GFTSMC achieves a faster convergence speed with a comparable control input, indicating that its control efficiency is higher.

**Figure 8:**
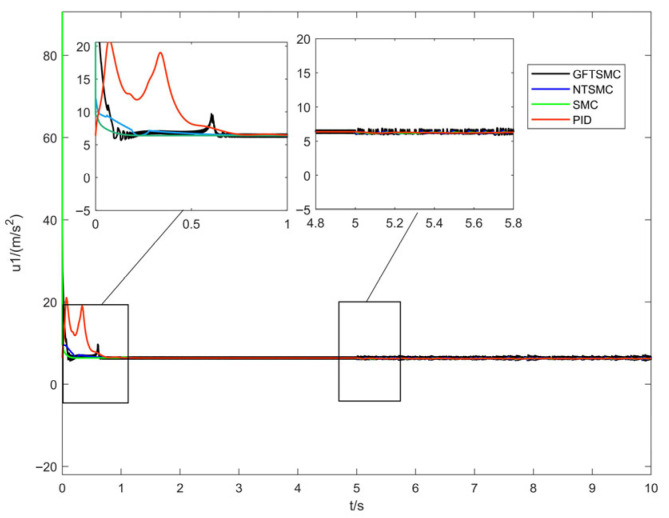
Response curves of total lift force.

Figure 8 presents the total-lift response curves of the quadrotor UAV under the four control methods. It can be observed that the lift amplitudes of the UAV across methods are similar; notably, the lift curve of the GFTSMC method does not exhibit significant abrupt changes after being subjected to disturbances, which indicates that the controller has excellent anti-disturbance capability.

The proposed GFTSMC demonstrates the following:

1. Rapid Convergence: 33.8–97.7% faster settling across all axes.
2. Precise Tracking: Minimal steady-state error even under disturbances.
3. Strong Robustness: Effective rejection of wind disturbances.
4. Practical Implementation: Smooth control inputs suitable for real applications.

These results validate GFTSMC as a superior control strategy for quadrotor trajectory tracking, balancing performance, robustness, and practical implementability.

## 5. Conclusions

This paper addresses the challenges of rapid response and disturbance rejection in quadrotor UAV trajectory tracking control. By employing a dual-loop decoupled control model and proposing a GFTSMC algorithm, we have developed an advanced trajectory tracking method. The simulation results demonstrate that GFTSMC enhances the rapidity of trajectory tracking control for quadrotor UAVs, strengthens their anti-disturbance capability in trajectory tracking control, ensures fast and stable tracking of the desired trajectory, and supports quadrotor UAVs in accomplishing operational tasks in scenarios demanding high real-time performance, high precision, and strong anti-disturbance.

Considering the increasing complexity of UAV missions, single-UAV operations may no longer meet the demands of emerging applications. Thus, our future research will focus on two key aspects: first, multi-UAV formation control, which involves extending GFTSMC to coordinated multi-agent systems, addressing the issues of inter-agent collision avoidance and formation maintenance, and enhancing swarm-level rapid response and disturbance resilience; second, experimental validation, which includes implementing the proposed algorithms on physical quadrotor platforms and conducting real-world field tests under various environmental conditions. These research directions are intended to advance the capabilities of UAV swarms in complex operational environments, especially for applications that require coordinated perception and action under uncertain conditions.

## Figures and Tables

**Table 1:** UAV parameters.

Parameter	Value
m	0.65 kg
g	$9.8\;m/s^{2}$
l	0.23 m
$I_{x}$	$7.5\times 10^{-3}\;kg\cdot m^{2}$
$I_{y}$	$7.5\times 10^{-3}\;kg\cdot m^{2}$
$I_{z}$	$1.3\times 10^{-2}\;kg\cdot m^{2}$
$I_{r}$	$6\times 10^{-5}\;kg\cdot m^{2}$
ρ	$3.1\times 10^{-5}\;N/{\left(rad/s\right)}^{2}$
γ	$7.5\times 10^{-5}\;N\cdot m/{\left(rad/s\right)}^{2}$

**Table 2:** Steady-state convergence time of the position loop and yaw angle.

Convergence Time/s	GFTSMC	NTSMC	SMC	PID
X-direction	0.13	0.47	0.60	2.63
Y-direction	0.64	1.00	1.40	2.14
Z-direction	0.12	0.30	0.37	0.93
yaw angle	0.047	0.071	0.074	2.047

**Table 3:** Maximum tracking error after introducing disturbances of the position loop.

Disturbance Error/mm	GFTSMC	NTSMC	SMC	PID
X-direction	0.038	0.378	2.62	14.06
Y-direction	0.072	2.18	4.98	22.94
Z-direction	0.022	0.13	0.81	7.49

## Data Availability

The data that support the study are available from the corresponding author, S.W., upon reasonable request. The data are not publicly available for privacy-related reasons.

## Abbreviations

The following abbreviations are used in this manuscript:

UAVs	Unmanned Aerial Vehicles
GFTSMC	Global Fast Terminal Sliding Mode Control
PID	Proportional–Integral–Derivative
SMC	Sliding Mode Control
NTSMC	Nonsingular Terminal Sliding Mode Control
